# Effects of the Extraterrestrial Environment on Plants: Recommendations for Future Space Experiments for the MELiSSA Higher Plant Compartment

**DOI:** 10.3390/life4020189

**Published:** 2014-05-05

**Authors:** Silje A. Wolff, Liz H. Coelho, Irene Karoliussen, Ann-Iren Kittang Jost

**Affiliations:** Centre for Interdisciplinary Research in Space (CIRiS), NTNU Samfunnsforskning AS, N-7491 Trondheim, Norway; E-Mails: liz.coelho@ciris.no (L.H.C.); irene.karoliussen@ciris.no (I.K.); a.i.kittang.jost@ciris.no (A.-I.K.J.)

**Keywords:** microgravity, magnetic field, radiation, roadmap, MELiSSA, plants, Moon, Mars

## Abstract

Due to logistical challenges, long-term human space exploration missions require a life support system capable of regenerating all the essentials for survival. Higher plants can be utilized to provide a continuous supply of fresh food, atmosphere revitalization, and clean water for humans. Plants can adapt to extreme environments on Earth, and model plants have been shown to grow and develop through a full life cycle in microgravity. However, more knowledge about the long term effects of the extraterrestrial environment on plant growth and development is necessary. The European Space Agency (ESA) has developed the Micro-Ecological Life Support System Alternative (MELiSSA) program to develop a closed regenerative life support system, based on micro-organisms and higher plant processes, with continuous recycling of resources. In this context, a literature review to analyze the impact of the space environments on higher plants, with focus on gravity levels, magnetic fields and radiation, has been performed. This communication presents a roadmap giving directions for future scientific activities within space plant cultivation. The roadmap aims to identify the research activities required before higher plants can be included in regenerative life support systems in space.

## 1. Introduction

Future missions to the Moon and Mars, involving long-term stays in space, rely on a life support system for food production and regeneration of resources. As identified through MELiSSA (Micro-Ecological Life Support System Alternative), such Closed Regenerative Life Support Systems (CRLSS) need to include a compartment for the production of higher plants [1,2,3,4,5]. Through CO_2_ absorption and O_2_ emission, water purification through transpiration, waste product recycling via mineral nutrition, and as a food source, plants play a key role in CRLSS [4,6,7]. On the Earth plants are known to adapt to extreme environments, and space experiments have demonstrated that plants are able to grow and reproduce in microgravity [8,9,10,11,12]. The first plant materials were brought into space in 1960, when seeds of wheat, pea, maize, and onion were flown on board of Sputnik 4 [13]. This was followed by photosynthetic measurements of *Chlorella* and the duckweed *Spirodela* [14] and with wheat seedlings and pepper plants on Biosatellite II [15]. Since then, a number of experiments have been successfully performed in a spacecraft, and a full life cycle of *Arabidopsis thaliana* has been completed on Salyut-7 [16]. The extensive effort and resources allocated to plant cultivation in space have revealed many answers, and also raised new research questions, especially with regard to food plants. Knowledge about the long term effects of the space environment on plant growth and development is essential for the design of a dependable CRLSS for space exploration beyond Low Earth Orbit (LEO).

The Literature Review of Higher Plants in Space for MELiSSA (LiRHiPliSME) project, contributing to MELiSSA, was initiated to analyze the present state of knowledge concerning the impact of space environments on higher plants. Focus has been on the effects on higher plants exposed to three factors on the Moon and Mars making the physical environment different from Earth: gravity levels, magnetic fields, and radiation [17,18]. The core activities in LiRHiPliSME have been a literature study, as well as a mobilization within the scientific community, including interviews with selected scientists and project workshops. Based on the LiRHiPLiSME project, and in collaboration with the European Space Agency, a roadmap giving directions for future scientific activities within MELiSSA and plant cultivation in space is presented.

## 2. Results and Discussion

### 2.1. The Physical Environment on ISS, the Earth, Moon and Mars

While the International Space Station (ISS) is in free fall, the Moon has 1/6, Mars 1/3 of Earth’s gravity. The strength of the Earth’s geomagnetic field is in the range of 30,000–60,000 nT [19], being strongest at the poles and weakest at the equator. The Moon and Mars have no global magnetic field, but only areas with local crustal magnetic fields that vary in strength and direction all over the surface [20,21,22]. On the Moon and Mars, the radiation levels are high, especially due to heavy ions from galactic cosmic rays (GCR) and energetic protons from large solar particles events (SPE). On the Moon’s surface the accumulated dose over the course of a year is about 57 cGy (=cSv) for GCRs and about 100 cGy per event for large SPEs. The accumulated dose on the Mars surface is 77 cGy per year for GCRs and 35 cGy per event for large SPEs. In comparison, the atmosphere and magnetic field surrounding the Earth provides radiation protection and the galactic GCR doses measured on Earth is 0.027 cGy per year and almost zero for SPEs [23,24]. The International Space Station (ISS) is located at low Earth orbit: here the radiation consists of GCRs and SPEs, and protons and electrons when passing through the South Atlantic Anomaly (SAA) of the radiation belt. The radiation dose at the ISS can vary but has been measured to be on average 15 cGy per year for GCRs, 4.6 cGy per year for SAA and up to 10 cGy within a few days during an intense SPE [25].

### 2.2. Main Conclusion from Literature Review

Plants have demonstrated their ability to grow and reproduce in space [8,9,10,11,16,17,26,27,28,11,16,17,26]. Although it has been documented that the reproduction phase does not depend on gravity for completion, the reproduction fitness is often reduced in Space and can cause a risk to the resource-use efficiency in plant based CRLSS [29,30]. Moreover, the influences of the space environment may result in an effect in the long term and over multiple generations, or have an impact on the plants’ role as food and part of a regenerative life support system. On the whole, the most frequently reported effects of a reduced gravity environment on plant physiology are secondary effects and linked to changes in the plants physical environment. This emphasizes the need for an advanced understanding of space effects on physiological transport and exchange, as well as adequate environmental control in the growth facilities for plant cultivation in space flight. Porterfield (2002) summarizes the biophysical limitations of gas exchange and physiological transport in the microgravity environment [31]. A brief overview of the established effects of gravity, radiation, and magnetic fields on higher plants is given below. More comprehensive presentations of the results from the literature review are published elsewhere [17,18].

#### 2.2.1. Gravity

Reduced gravity environments influence the plants physical environment that again affects the physiological transport of water and solutes, and gas exchange between the plant and its surroundings [31]. These effects are called indirect effects of gravity because they are not caused by gravity interacting with the mass of the plant body itself. As an example, the lack of buoyancy driven thermal convection (BDTC) in microgravity and the consequent increase of boundary layer thickness, causes biophysical limitations on the processes of gas exchange and transpiration in higher plants [31]. In the aerial plant parts this effect can be diminished by proper ventilation and forced air movement [10,32]. In the root zone the problem is more complex, and root zone hypoxia induced by gravity dependent changes in fluid and gas distribution remains a persistent challenge for plant experiments in microgravity [33,34,35]. Diffusion limited gas exchange and root zone hypoxia can result in a reduced uptake and transport of nutrients in plants [31]. Some studies indicate that the stunted growth observed in microgravity can be linked to nutritional issues [36,37] and that nutrient uptake is altered by the space environment [38,39]. The results of these studies [36,37,38,39] are challenging to interpret due to the limited information on hardware, experimental set up, degree of environmental control and ventilation in the growth chambers. When looking at changes in plant medium composition after a spaceflight experiment in NASA’s Plant Growth Unit, Levine and Krikorian (2008) found a reduced amount of potassium in the spaceflight exposed growth medium indicating an elevated potassium uptake in plants grown in space [40]. This was argued to be either an increased quantity of root tissue, or to a microgravity related change in uptake kinetics. Another study showed no differences in nutrient uptake rates between ground and flight exposed plant material in ventilated chambers [41]. Thus, studies on the effects of the space environment on plant nutrition are inconclusive and very limited, and no study has, as far as we know, assessed effects on the rhizosphere.

Plant gas exchange, metabolism, and photosynthesis mechanisms were not affected by microgravity when provided with satisfactory environmental control [26,42]. A reduction in the activity of the photosystem activity has been reported [42,43]. Still, more studies are required to draw a final conclusion about the potential effects of reduced gravity on photosynthesis. A research based understanding of the influence of gravity on physiological transport and exchange will enable hardware technology development and technological solutions to overcome these challenges.

Spaceflight experiments reveal no detrimental impact of gravity or other space factors on the morphology of higher plants in either short or long term flights (one generation period). One of the best characterized gravity responses of plants is the directed growth in response to gravity, called gravitropism [44]. The extensive work on gravitropism, including space experiments, is reviewed in several articles [45,46,47,48,49,50]. Studies of lentil roots have documented automorphogenesis and autotropism under microgravity conditions [51]. Higher plants respond to a range of environmental stimuli in addition to gravity; for example light (phototropism) and water (hydrotropism [52]). Under microgravity conditions the plant will still orient according to the light source and water potential gradient [53,54]. These responses are not necessarily the same as on ground, e.g., *Arabidopsis* hypocotyls responds with an increased blue-light phototropic reaction under microgravity compared to a 1 g control [53]. Influence of gravity has also been observed on the ultrastructure of cell organelles, e.g., larger chloroplasts and randomly distributed amyloplasts, in addition to a thinner cell wall combined with a decrease of cell wall constituents (polysaccharides) [55,56,57].

#### 2.2.2. Radiation

Cosmic radiation alter gene expression levels and affect the genome through DNA damage and chromosome mutations [58]. At this point, however, the effects do not seem to be detrimental for plant growth and survival [18]. Still, and despite the fact that plants have been grown in low Earth orbit during several consecutive generations [27], it is still not known if the plant genome will remain stable under space conditions.

Due to the shielding of the experiment facility, which is a prerequisite for humans in manned space exploration, long term exposure to low chronic radiation is considered to be more relevant than high acute radiation doses. Moreover, chronic exposure to low doses of ionizing radiation has been shown to have a comparatively stronger influence on plant genetics than an acute dose [59]. Rather few studies have been performed with chronic radiation exposures [60]. Chronic exposure to ionizing radiation seems to affect the genetic structure of populations in the long term, and a reduction of genetic variability may be an adaptive process associated with chronic stress [61]. Different mechanisms are involved in the response to chronic or acute exposure to radiation [62]. While the most well represented group of genes affected by acute radiation exposure is a group of oxidative stress-related genes, chronic stress leads to a totally different response that reflects in adaptive responses by regulating genes belonging to general stress and nucleic acid metabolism. Chronic stress also induces several genes involved in photosynthesis and carbohydrate metabolism [62]. Chronic exposure with different levels of low-dose gamma radiation causes a reduction in fresh weight of roots, stems and leaves of *A. thaliana*, without discernibly affecting oxidative stress pathways [63]. This supports previous results [59,62]. Different species show varying resistance to radiation damage [64]. Consequently, the experiments with radiation on ground should focus on low chronic radiation exposure and different species including food plants.

#### 2.2.3. Magnetic Fields

The most significant role of the Earth’s magnetic field is to provide shielding from space radiation. Since there is no global magnetic field on Moon and Mars, tests with plants exposed to very weak magnetic shields are important. There are studies indicating that a magnetic field lower than the geomagnetic field directly causes changes in plant growth and development [18,65], and plant metabolism [66,67], in some cases by inhibition, in other cases by enhancement. In contrast, several studies have been performed with a magnetic field on top of the geomagnetic field indicating an influence on plant growth and photosynthesis [68,69,70,71,72,73,74,75]. Both kinds of studies suggest that changes in magnetic fields might impact plant growth and development. A recent study indicates that plants through evolution have developed a magneto receptor mechanism where the plant cryptochrome is central [76]. Even though the necessity of these experiments has been stressed [77], magnetic field experiments in space have not been performed so far, only experiments on ground have been reported.

## 3. Directions and Requirements for Future Research

In general, the primary objectives for future research activities should be linked to the fundamental processes required to ensure sustainable plant production in space, *i.e.*, effects of the space environment on the processes of photosynthesis, gas exchange, transport of water and solutes and stability of the plant genome. Experiments should also, whenever feasible, include assessment of a plant’s complete growth cycle. Since the hardware has turned out to be of great importance for the results in microgravity research, an optimized experimental design with full environmental monitoring and control must be the standard for future experiments. This includes a detailed description of climatic conditions and protocols for sowing, plant handling and analysis. Selecting a few model plants, including crop plants for life support, would further increase the comparability between studies. There is a consensus worldwide that preferred characteristics for CRLSS crops are a short cultivation cycle from seed to seed, high productivity and resistance against pathogens, reduced plant size, high levels of adaptability to expected space conditions, and stress tolerance [4]. In addition, cultivars with high nutritional value for astronauts and low levels of anti-nutritional factors and non-edible biomass (waste) are favoured. For MELiSSA, four crops high in energy and/or protein have been selected: bread wheat, durum wheat, potato, and soybean [4].

International collaboration between space agencies, both during experiment preparation and implementation, and through sharing of experiences and results would increase the output of space plant experiments [78].

### 3.1. Higher Plant Model

A valuable tool in characterising and understanding the plant physiological processes under space conditions is the development of mathematical models. A proper model for plant physiological processes should include the complex interplay between environmental, physiological, biophysical and bio mathematical factors [79]. Hezard *et al.* [80] developed a model that separates the different plant organs in order to study the various sub-processes. All these sub-models fit into a generally structured model predicting the CO_2_ and nutrient solution consumption, as well as the oxygen, clean water, and food production of the MELiSSA higher plant compartment in different environmental conditions [80]. Another model describes the mass flux at the surface of the plant leaf in a life support system [81]. This includes transport phenomena, such as the vaporisation of water, CO_2_ uptake, oxygen release, and respiration. Impact of the external environment is included in the model, even for gravity levels on the Moon and Mars [81].

To increase the predictability of the higher plant compartment, the MELiSSA program pre-flight activities include extensive food characterisation studies on ground to fully describe and understand the chosen MELiSSA species and all the processes related to them [4,29,82,83]. The food characterisation studies will support the development of a multi-parameter model termed the Higher Plant Model (HPM), which will describe the physiological processes in the higher plant chamber. The HPM must be validated under space conditions, especially the parameters known to be affected by gravity like physiological transport and exchange.

### 3.2. Ground Based Experiments

After an evaluation of the available technical solutions for simulation of space conditions, chronic exposure to low radiation seems to be the most realistic variable to be assessed in pre-flight experiments. Priority should be given to effects of radiation on biomass production, photosynthesis, and gas exchange, gene expression profile, along with all processes affecting the plants nutrient value. Subsequently, it is important to consider the effects of chronic irradiation on morphological changes, chromosome aberrations and mutation frequency, since these are good measures of plant development and genome stability. The radiation exposure should mimic space radiation as much as possible, and include at least gamma-rays, proton and neutron particles. Existing facilities for radiation experiments are the Radioactive Isotope Beam Factory RIKEN (Nishina Center for Accelerator-Based Science, RIBF), the HIMAC (Heavy-Ion Medical Accelerator in Chiba), both located in Japan, as well as the Alternating Gradient Synchrotron in Brookhaven, USA. The available facilities for radiation experiments and simulation of space conditions will only partly simulate the whole radiation load in space, even though a large number of rays and particles with high energies can be obtained. The two-dimensional (2D) clinostats and random positioning machines (RPM) are widely applied and useful methods for simulation of microgravity (reviewed by van Loon [84]), but the spatial dimensions are strongly restricting the sample size and cultivation method. Another ground based method for simulating microgravity is magnetic levitation, using a vertical bore magnet for levitation of biological material [85]. In a comparative study of the different methods for microgravity simulation, magnetic levitation was found to be of limited use due to the inability to levitate plant gravisensors (statholites), and more generally because of the difficulty in separating the effects of levitation from other effects of the strong magnetic field on the organism [85]. Thus, the available platforms for microgravity simulation are valuable tools for basic research but were evaluated by the LiRHiPliSME team and project partners to be insufficient for the study of food plants and long term higher plant experiments for life support applications.

In future life support systems with higher plants, a recirculating hydroponic system is considered to be the preferred cultivation system. To achieve the required process control in such a system, extensive research is required on basic plant nutrition and rhizosphere processes. In parallel with the scientific investigations, development of sensor technology for surveillance of nutrients in the solution and plant nutrient status should take place. The optimal solution would be a system with a high recycling capacity and real time surveillance of plant nutrient and water status.

### 3.3. Space Experiments

Water and nutrient management is considered to be one of the most challenging aspects of plant cultivation in space, and there is a need for both scientific activities and technology development [31,86]. The effects of gravity on basic physical phenomena of all matter, and how these effects in turn influence on the biological system, need to be elucidated before the direct effects of gravity on the cell, tissue, organ, or whole organism can be revealed [33]. It should be distinguished, however, if the final goal is plant cultivation in microgravity (like in Low Earth orbit or on an asteroid), or on the surface of the Moon or Mars with fractional gravity present.

Based on reported results from space experiments, several issues related to potential effects of fractional- or microgravity on food quality and safety need to be examined. These should for example include changes in the cell wall components [55,56,87,88,89] and changes in secondary metabolite production [90,91,92]. Thus far, radiation effects have not been measured or discriminated from potential microgravity effects in any of the reported plant experiments in low Earth orbit. One way to separate these effects is an in-flight reference centrifuge providing a 1× *g* gravity environment while all other factors of spaceflight are the same. Centrifuges also allow for experiments with Mars (0.38× *g*) and Lunar (0.17× *g*) gravity exposures, and whether or not higher plants can sense gravity in these environments [5]. The importance of this kind of control has been stressed and is realized in several facilities available for plant research on the ISS, like Biolab and the European Modular Cultivation System (EMCS) [28,93,94,95,96]. Including a dosimeter in the growth chambers would accurately measure the amount of radiation received by the plants.

Experiments on ground with shielding from the Earth’s magnetic field show that weak magnetic fields influence plant gas exchange and metabolism. However, more experiments are required to ascertain the effect of magnetic fields, especially for the growth conditions on the Moon and Mars. To assess the effects of total space radiation load, and potentially in the absence of a magnetic field, experiments need to be performed outside low Earth orbit, e.g., as being part of robotic missions to the surfaces of the Moon and Mars. For these missions the return of plant material to Earth for analysis is very limited, and permanent supervision of the plant’s growth and health status preferably on the basis of remote sensing technology, is required.

Today, the best site for performing space experiments with higher plants is the ISS. Satellites, and to some extent parabolic flights, are good alternatives to analyse the short term effects of fractional gravity on photosynthesis and physiological transport, especially gas exchange and transpiration. Longer exposure to space conditions can be studied in low Earth orbit on the ISS with facilities like EMCS. These facilities allow experiments with full environmental control while exposing the plants to the gravity conditions of Moon and Mars.

### 3.4. Roadmap

**Figure 1:**
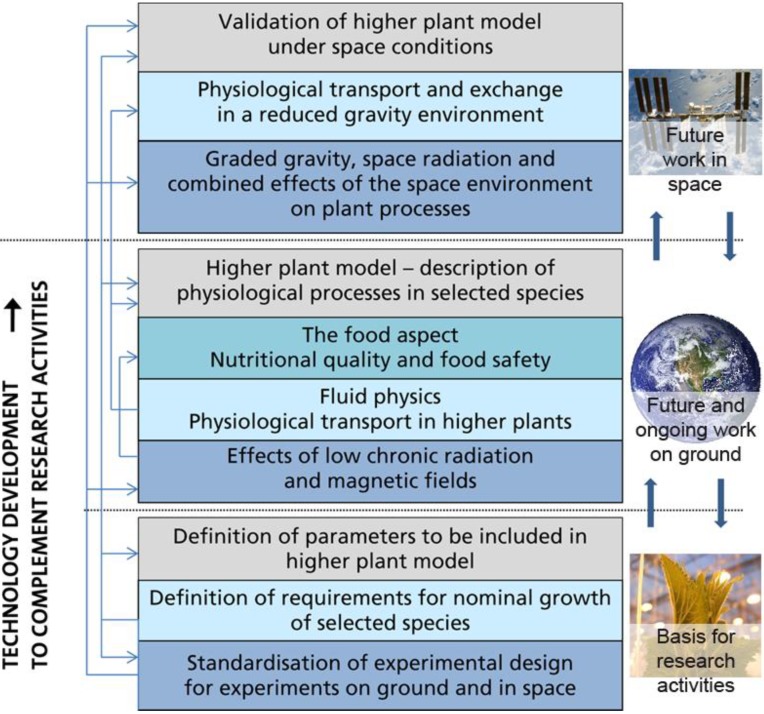
Main groups of requirements for future space research activities on higher plants. The lower section of the describes activities to be performed as a basis for future research activities, the middle section describes pre-flight experiments, while the upper section of the figure describes space experiments required to reach the scientific readiness to develop regenerative life support systems containing higher plants.

Based on the LiRHiPliSME project and directions given above, the research activities required to reach the scientific readiness for further development of a CRLSS containing higher plants have been grouped into a set of building blocks. These building blocks, forming the basis for the roadmap, are presented in Figure 1.

**Figure 2:**
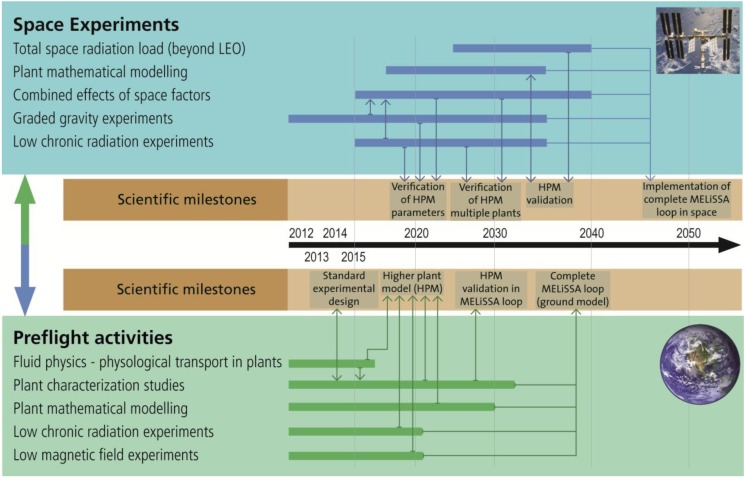
Roadmap for future research activities on higher plants as part of a life support system for space exploration. The lower section describes preflight activities to be performed on the ground, while the upper section describes future plant related research activities in space. The food characterization studies are carried out as preflight activities to characterize the species-specific qualities of the chosen MELiSSA crops (*i.e.*, growth, development and metabolism), and will provide input to the development of the HPM.

In Figure 2, the building blocks are placed in a timeline, forming a roadmap assuming implementation of a complete MELiSSA life support system with higher plants operating in space by 2050. The roadmap aims to outline the scientific activities leading to milestone achievements towards sustainable plant growth and food production in space. Ground based activities include the development of mathematical models and food characterisation studies (describing the plants growth, development and metabolism) aiming to fully characterize and understand the chosen crops and all the processes related to them. Plant experiments beyond LEO are envisaged as part of a manned mission. The roadmap presented is limited to realization of a higher plant chamber as part of the MELiSSA loop, and the references to the complete MELiSSA loop are included for programmatic clarity.

## 4. Conclusions

Extensive research performed within space plant biology have enabled us to conclude that higher plants are able to adapt to space conditions in low Earth orbit, at least during one generation from seed to seed. However, sufficient environmental control, including forced ventilation, trace gas control and a well-functioning system for water and nutrient delivery, is required for successful experiments. The information about experiments dealing with whole plant physiology in microgravity is limited, and the long term effects of space conditions, especially outside LEO, need to be better characterized.

Before the successful integration of higher plants into a CRLSS can take place, more data are required to determine long term effects on fundamental plant processes after chronic exposure to radiation, to a weak magnetic field and to fractional gravity. Physiological transport and exchange, both within the plant, and between the plant and its environment, should be prioritized as it impacts plant metabolism and is affected by gravity. The rootzone and rhizosphere requires special attention. A valuable tool in early stress detection and understanding plant responses to space conditions is the development of mathematical models describing the expected metabolic pattern for the species being studied under “normal” or Earth conditions.

Both, the new technology emerging from the process towards CRLSS and the development of crop models can be regarded as the applied aspect in space research, compared to the more fundamental research using non-edible model plants, such as *Arabidopsis thaliana*. CRLSS have strong synergies to sustainable agriculture and food production on Earth, which is an aspect of high priority in the science community and the society in general. Implementing a better coordination between the applied and the fundamental research communities is believed to improve the scientific results both in quantity and quality and, thus, maximize the use of the resources linked, e.g., to the ISS platform and the ground based facilities.

## Abbreviations

BDTC: Buoyancy Driven Thermal Convection
CRLSS: Closed Regenerative Life Support System
EMCS: European Modular Cultivation System
GCR: Higher Plant Model
HPM: Galactic Cosmic Rays
ISS: International Space Station
LEO: Low Earth Orbit
LiRHiPliSME: Literature Review of Higher Plants in Space for MELiSSA
MELiSSA: Micro-Ecological Life Support System Alternative
RPM: Random Positioning Machine
SAA: South Atlantic Anomaly
SPE: Solar Particle Event
